# Investigating the impact of the environment on neurodevelopmental disorder

**DOI:** 10.1186/s11689-020-09345-y

**Published:** 2020-12-16

**Authors:** Heather Volk, Margaret A. Sheridan

**Affiliations:** 1 Departments of Mental Health and Environmental Health and Engineering, Bloomberg School of Public Health, Johns Hopkins University, 624 N. Broadway, HH833, Baltimore, MD 21205 USA; 2 Intellectual and Developmental Disabilities Research Center, Kennedy Krieger Institute, Baltimore, USA; 3 Department of Psychology and Neuroscience, University of North Carolina, 238 E. Cameron Street, Office 248, Chapel Hill, NC 27599 USA; 4 Carolina Institute for Developmental Disabilities at the University of North Carolina, Chapel Hill, Chapel Hill, USA

How do we define the environment? In this special issue of the *Journal of Neurodevelopmental Disorders* (JNDD), we take a broad view, publishing research linking psychosocial adversity to risk for neurodevelopmental disorders side by side with studies examining the effects of exposure to environmental toxicants, such as air pollution and heavy metals. This issue both reflects the scope of the conceptualization of the ‘environment’ and the breadth of methods used to investigate it with studies spanning preclinical research, clinical studies of patients, and epidemiological approaches. Each of these studies reflects research pursued within one of 14 Intellectual and Developmental Disabilities Research Centers (IDDRCs) and showcases the breadth and depth of work these centers support.

The link between psychosocial risk early in life and negative neurodevelopmental outcomes has been observed for decades. Increasingly research has documented the impact of these experiences on central nervous system function and structure while randomized control trials have revealed that some of these associations are driven by causal linkages between early psychosocial adversity and neural outcomes [1, 2]. At the same time, the field documenting the impact of environmental toxicants on neurobiology and health outcomes has also grown dramatically in recent years. Both large scale epidemiologic studies and animal models have revealed the long-term impact of early exposures to air pollution, pesticides, heavy metals, and malnutrition. Thus, recent research has revealed that multiple kinds of exposures are likely to impact the development of the function and structure of the central nervous system, yielding a common pathway which may transduce multiple kinds of risk into impairments in cognition, social processing, and emotional functioning that manifest as neurodevelopmental disorders. Importantly, exposure to both psychosocial and environmental risk is often differential by social class and race, concentrating exposure to both forms of risk in a specific population. Considering the developmental environment broadly including examining psychosocial adversity, environmental toxicants, and malnutrition in studies that are in conversation with each other can open up new avenues of discovery. This work can identify shared pathways leading to risk for neurodevelopmental disorders across exposure or reveal potential cross-exposure interactions. In addition, this concentration of risk is a call to action, focusing on the need to address the issue of environmental justice more broadly than in relation to the chemical environment.

The research in this issue spans multiple levels of analysis, from animal models to population-based studies to community level investigations—all with the goal of addressing the role of the environment, broadly defined. With regard to psychosocial adversity, Smith and Pollak contribute a review highlighting the link between psychosocial adversity (prenatal and postnatal stress) and developmental changes in neurobiology (Smith and Pollak, *this issue*). This paper considers many psychosocial risk factors together. In contrast, Humphrey and colleagues elegantly link exposure to deprivation, a specific psychosocial adversity, to receptive language. This two-sample study spans degrees of exposure and draws a line connecting two forms of psychosocial adversity, institutionalization, and low socioeconomic status, by identifying the common experience of deprivation within both exposures (Humphreys et al., *this issue)*.

Other forms of environmental adversity are explored in subsequent papers. Several contributions included here seek to provide biological evidence of air pollution and pesticide exposures from mouse models (Silverman et al., Cole et al., Hashimoto-Torii et al., *this issue*) to neurodevelopmental outcomes. These animal studies are juxtaposed with population-based studies of prenatal air pollution exposure and maternal immune response on ASD risk and low-level lead exposure on cognitive ability (Volk et al., Mazumdar et al., *this issue*). In a review, Cristancho and Marsh highlight the role that epigenetics may play in linking prenatal hypoxia exposure to neurodevelopmental disorders identifying a potential final common pathway for many forms of environmental risk (Cristancho and Marsh, *this issue*). Finally, McGrath and colleagues describe an ecological analysis of sociodemographic factors and non-native English language speaking on ASD rates in New York state (McGrath et al., *this issue*). This final paper, which uses sociodemographic variables that cluster other forms of risk as a predictor, highlights that at the population level, many aspects of the environment may work together to increase risk for neurodevelopmental disorders.

Across these studies, a common theme emerges, the impact of the environment, broadly construed, on the development of the central nervous system. What remains to be done is linking across these important areas of investigation, identifying the clustering of risk across the chemical and social environment, observing final common pathways impacted by multiple forms of environmental exposures, and most importantly considering the ways in which these forms of risk interact.

Together, this collection of research illustrates that we cannot view intellectual and developmental disabilities narrowly. Investigations from the IDDRCs, and beyond, are essential for fostering improved understanding of not only the risk for onset, but also impact on the trajectory of impairment, for individuals with intellectual and developmental disabilities. The work reported on herein displays the strengths of the individuals IDDRCs as well as the collective strength of this network in integrating multiple levels of analysis and areas of expertise that are a hallmark of these inter-disciplinary centers. Fostering such within and cross-institutional expertise in both the study of the environment and neurodevelopment is essential for improving the health and well-bring of affected individuals and their families.

## Data Availability

N/A
